# Strengthening the migrant-friendliness of Thai health services through interpretation and cultural mediation: a system analysis

**DOI:** 10.1186/s41256-020-00181-0

**Published:** 2020-12-08

**Authors:** Hathairat Kosiyaporn, Sataporn Julchoo, Mathudara Phaiyarom, Pigunkaew Sinam, Watinee Kunpeuk, Nareerut Pudpong, Pascale Allotey, Zhie X. Chan, Tharani Loganathan, Nicola Pocock, Rapeepong Suphanchaimat

**Affiliations:** 1 International Health Policy Program, Ministry of Public Health, Nonthaburi, Thailand; 2 United Nations University – International Institute for Global Health, Kuala Lumpur, Malaysia; 3 Centre for Epidemiology and Evidence-based Practice, Department of Social and Preventive Medicine, Faculty of Medicine, University of Malaya, Kuala Lumpur, Malaysia; 4 Department of Global Health and Development, Faculty of Public Health and Policy, London School of Hygiene and Tropical Medicine, London, UK; 5 Division of Epidemiology, Department of Disease Control, Ministry of Public Health, Nonthaburi, Thailand

**Keywords:** Migrant health worker, Migrant health volunteer, Interpreter, Cultural mediator, Thailand

## Abstract

**Background:**

In addition to healthcare entitlements, ‘migrant-friendly health services’ in Thailand include interpretation and cultural mediation services which aim to reduce language and cultural barriers between health personnel and migrants. Although the Thai Government started implementing these services in 2003, challenges in providing them still remain. This study aims to analyse the health system functions which support the interpretation and cultural mediation services of migrant health worker (MHW) and migrant health volunteer (MHV) programmes in Thailand.

**Methods:**

In-depth interviews were conducted in two migrant-populated provinces using purposive and snowball sampling. A total of fifty key informants were recruited, including MHWs, MHWs, health professionals, non-governmental organisation (NGO) staff and policy stakeholders. Data were triangulated using information from policy documents. The deductive thematic analysis was classified into three main themes of evolving structure of MHW and MHV programmes, roles and responsibilities of MHWs and MHVs, and supporting systems.

**Results:**

The introduction of the MHW and MHV programmes was one of the most prominent steps taken to improve the migrant-friendliness of Thai health services. MHWs mainly served as interpreters in public facilities, while MHVs served as cultural mediators in migrant communities. Operational challenges in providing services included insufficient budgets for employment and training, diverse training curricula, and lack of legal provisions to sustain the MHW and MHV programmes.

**Conclusion:**

Interpretation and cultural mediation services are hugely beneficial in addressing the health needs of migrants. To ensure the sustainability of current service provision, clear policy regulation and standardised training courses should be in place, alongside adequate and sustainable financial support from central government, NGOs, employers and migrant workers themselves. Moreover, regular monitoring and evaluation of the quality of services are recommended. Finally, a lead agency should be mandated to collaborate with stakeholders in planning the overall structure and resource allocation for the programmes.

## Introduction

The health of migrants is recognised as a global health issue in several international agreements [1, 2]. International migration is increasing (from 2.8% of the total global population in 2000 to 3.5% in 2019), and migrants face particular health challenges as a socially excluded group, including difficulties in accessing healthcare where they experience legal, financial, language, cultural and informational barriers [2, 3]. Within the Southeast Asia (SEA) region, figures from mid-2019 showed that international migrants accounted for 10.2 million or 12.2% of all migrants in Asia [3]. The International Organization for Migration (IOM) Migration Data Portal showed that Thailand ranked first as a preferred destination, receiving 30% of migrants in SEA [3]. As of 2019, there were an estimated 3.6 million migrant workers in Thailand, a three-fold increase from 1.3 million in 2000 [4]. Migrant workers from Myanmar, Lao PDR, Cambodia and Vietnam, who are employed mainly in the construction, agriculture and fishing sectors, comprise nearly 10% of the entire labour force in Thailand [5]. Accordingly, migrant health gains significant attention from the Thai government since protecting the health of migrants means ensuring the protection of the Thai economy and the right to health, though at the same time this needs to be balanced with the concern of national security if the country accepts too many inbound migrants and allows them to have relatively similar rights with the Thai nationals [6].

The Migrant Integration Policy Index (MIPEX) has carried out a benchmarking of migrant integration policy in 38 countries in Europe and beyond. The Health strand assesses four dimensions of policy: entitlements to health services; accessibility of services; responsiveness to migrants’ needs; and measures to achieve change [7]. With regard to migrants’ entitlements to health services, in 2014 the Thai government introduced the One Stop Service (OSS) in order to include all undocumented migrants in the health system [6]. After passing the national verification process, documented migrants must pay for health insurance – Social Security Scheme (SSS) for formal workers and Health Insurance Card Scheme (HICS) for informal workers and their dependants [6].

Policies to improve the accessibility of health services include the provision of health education, health promotion, information about services and ‘cultural mediators’. Responsiveness to the needs of migrant patients includes providing qualified interpretation services and promoting ‘cultural competence’ or ‘diversity sensitivity’ [7]. Cultural competence aims to bridge gaps between health workers and patients with a migrant background [7, 8]. It can be promoted by ensuring culturally diverse staff, providing staff with appropriate training, and adapting signage and instructional literature to migrants’ languages and cultural norms [9]. In Thailand, the Ministry of Public Health (MOPH) provided a multilingual medical dictionary and basic communication courses for health personnel, to promote better communication with migrants [10, 11]. Additionally, multilingual signs and leaflets were made available at health facilities [12].

Culturally competent healthcare services enhance the accessibility and quality of services by reducing language and cultural barriers. Klemm et al. (2016) described the distinction between linguistic interpretation and cultural mediation. Interpretation involves conveying the meanings of language during interpersonal interactions as accurately as possible; it is a professional activity, with recognised standards and codes of conduct [13]. Cultural mediation is about bridging ways of thinking and non-verbal communication between health workers and migrants, giving both parties deeper insight into each other’s meanings and cultures in order to foster mutual understanding [14]. Cultural mediators help health professionals understand and be aware of the cultural practices of migrants, as well as informing migrants about their entitlements and helping them navigate the health system [14]. In some countries, these two roles overlap, e.g. interpreters in Belgium do not limit themselves to only linguistic translation but also provide health-related information to migrant patients [13]. In Thailand, the term ‘cultural mediator’ includes the roles of health service navigator, health coordinator and health educator.

Since 2003, ‘migrant-friendly health services’ have been implemented under a collaboration between the Thai MOPH and non-governmental organisations (NGO) in migrant-populated areas [12]. Services that were developed include the migrant health worker (MHW) and migrant health volunteer (MHV) programmes reviewed in this paper [12]. These programmes were designed to reduce language and cultural barriers among migrants, leading to improved access to healthcare and health outcomes [15, 16]. MHWs are assigned to be interpreters, health assistants and coordinators, and health educators in health facilities and migrant communities. MHVs have the roles of health assistants and coordinators, and health educators only in migrant communities [15, 16]. Some activities may be carried out by both MHWs and MHVs. For instance, if MHWs are not available, some MHVs may step in to assist health professionals in communicating with and providing health services to migrant workers. Therefore, interpretation and cultural mediation proficiencies are important for both MHW and MHV roles.

According to the World Health Organization (WHO), health system building blocks are as follows: service delivery; health workforce; information; medical products, vaccines and technologies; financing; and leadership and governance (stewardship) [17]. All of these blocks contribute to a framework that can identify capacity gaps and key priorities, as well as setting the desirable attributes of a health system [16]. To deliver effective MHW and MHV programmes, these programmes should have qualified human resources, adequate health financing and good governance. Therefore, this study uses these three functions as a framework for system analysis.

Previous studies evaluating the MHW and MHV programmes have outlined the following constraints: low numbers of MHWs and MHVs, limited interpretation skills, lack of training programmes and supervision, and lack of budgetary support [18, 19]. Up to now, no study has examined the interpretation and cultural mediation services provided by the MHW and MHV programmes in Thailand, particularly regarding the issue of operational constraints and the challenges of delivering migrant-friendly services. This study aims to describe the Thai health system functions that support the interpretation and cultural mediation services of MHW and MHV programmes.

## Methods

### Research design and participants

A qualitative approach was employed using in-depth interviews triangulated with a policy document review. Purposive sampling was used to identify key informants in both public and non-governmental organisations. Additional informants were identified by snowball sampling. The study took place in the two most densely migrant-populated districts in Samut Sakhon and Ranong provinces, Thailand. Key informants included MHWs, MHVs, health professionals, NGO staff and policy stakeholders.

### Measurements and data collection

Ethical approval to conduct the study was obtained from the Institute for Human Research Protection, Thailand (IHRP 530/2561). The interview topic guides consisted of three parts: a) background and structure of the interpretation and cultural mediation services in MHW and MHV programmes; b) health system support for these programmes focusing on human resources, financial management and inter-agency coordination; and c) strengths and weaknesses of the system and recommendations for the services. Each interview took about 30–45 min and was recorded with consent from the interviewees. All interviews took place in the households or workplaces of key informants from November 2018 to April 2019.

### Data analysis

The interviews were transcribed from the audio records and coded based on themes. A deductive thematic analysis was conducted to identify three main themes: 1) evolving structure of MHW and MHV programmes, 2) roles and responsibilities of MHWs and MHVs, and 3) supporting systems in need. The supporting systems were classified into three sub-themes of budget, human resource development, and inter-agency coordination and planning for MHW and MHV programmes.

## Results

There were 50 key informants participating in this study (see Table 1). Most of them were female (female = 32; male = 18). In term of work responsibilities, there were 18 health professionals, 12 MHWs, 9 MHVs, 7 policymakers and 4 representatives from NGOs.

**Table 1:** Characteristics of key informants

Acronym	Participants’ background	Key informants (N)	Sex	
			Male	Female
MHW	Migrant Health Workers	12	5	7
MHV	Migrant Health Volunteers	9	1	8
HP	Health Professionals	18	6	12
NGO	Non-Governmental Organisations	4	2	2
POL	Policy Stakeholders	7	4	3
	**Total**	**50**	**18**	**32**

We identified three main themes from the interviews: 1) evolving structure of MHW and MHV programmes, 2) roles and responsibilities of MHWs and MHVs, and 3) supporting systems in need.

### Evolving structure of MHW and MHV programmes

Originally, before 1995, interpretation services for migrant workers in Thailand were primarily provided by MHVs from NGOs; their voluntary work mainly involved interpretation and other kinds of help with overcoming language barriers experienced by migrants. Afterwards, they were employed as MHWs because there was a need for interpreters, especially in public health facilities and NGOs. These services were subsequently scaled up with technical and financial support from the IOM, the United States Agency for International Development (USAID) and the Thai MOPH [12]. This ‘Migrant Heath Programme Model’ was piloted in two provinces, then further expanded to five provinces [12]. In 2008 the donors withdrew their financial support, leaving local NGOs and the MOPH to continue the project [12]. To sustain the programme, the MOPH provided support mostly for training materials and guidelines, with limited funding to supplement local organisations’ financial and human resource development. Separately, NGOs have developed MHW and MHV programmes aligned with MOPH training guidelines.

*“Since 1995, our organisation [NGO] was involved in migrant health in province B. There was a language barrier because we did not have staff who could speak Burmese, so we had to hire Myanmar doctors in our team. The doctor salary was too high, so we could employ only one or two doctors which was not enough for our workload. Therefore, we tried to hire more staff called the ‘Frontline Social Network’ [MHWs] who networked with the target group [migrant workers] in this area.” [NGO-4].*

### Differentiation between MHWs and MHVs

MHWs are hired as staff in public health facilities and with NGOs in migrant communities, while MHVs work as volunteers within migrant communities. The MOPH training guidelines include content that emphasises differing roles and responsibilities of MHWs and MHVs (see Table 2). The common tasks of MHWs and MHVs are to provide health education, assist health staff and coordinate interactions between health staff, migrant communities and other agencies [15, 16]. Responsibilities of MHWs have expanded beyond those of MHVs, including translating bilingual materials, joining training courses and meeting regularly, surveying migrant demographic data in communities and following up home health care [15, 16].

**Table 2:** Roles and responsibilities of MHWs and MHVs

Components	Migrant health workers	Migrant health volunteers
**Workplaces**	Health facilities and communities	Communities
**Allowances**	Yes	No
**Roles and responsibilities**		
• Interpretation	Yes	No
• Providing health education e.g. health insurance registration, health promotion and disease prevention	Yes (including to MHVs)	Yes
• Coordinating among health staff, migrant communities and other agencies, e.g. reporting disease outbreaks	Yes	Yes
• Assisting health staff, e.g. screening diseases	Yes	Yes
• Being role models of healthy lifestyles	Yes	Yes
• Translating bilingual materials	Yes	No
• Joining training courses and meeting regularly	Yes	No
• Surveying migrant demographic data in communities	Yes	No
• Following-up home health care	Yes	No

Source: Training Curriculum for Migrant Health Workers (2016) [15] and Training Curriculum of Migrant Health Volunteers (2016) [16]

In general, MHWs in public health facilities are involved mostly with interpretation functions while MHWs working in communities are responsible for additional tasks. These include being health assistants and cultural mediators (i.e. health service navigators, health coordinators and health educators). MHWs also have to help with disease screening (e.g. measuring blood pressure); coordinating interactions between and with health professionals; and educating MHVs and migrant workers about basic measures like personal hygiene and reproductive health [15, 16]. At the beginning of the programme, MHVs were deployed as interpreters only. When the MHW programme started, the role of MHVs then expanded to include cultural mediation in migrant communities. Although MHWs are formally assigned as interpreters, MHVs are in practice deployed as interpreters in situations where MHWs are not available. Nevertheless, not all MHVs are able to offer interpretation support. The roles of MHWs and MHVs vary depending on the organisational context and individual capacities.

*“The key roles of MHW [in public health facilities] is providing interpretation services and partly assisting with nurse aides such as cleaning rooms, recording blood pressure or circulating patient records.” [HP-10].*

*“We [NGOs] do not expect that MHVs can do everything, but we expect at least one function such as being interpreters, health educators, advocate for health issues or coordinators.” [NGO-2].*

*“Some [migrant workers] came [to health centre] with stomach ache … when I asked more about the characteristics of pain, they could not answer … then I called MHVs to interpret” [HP-8].*

### Systems analysis of MHW and MHV programmes

Various mechanisms are involved in the sustainability of MHW and MHV programmes in Thailand, such as laws and regulations supporting the formal status of MHWs, and budgets for employment and human resource development. Through system analysis, we identified several challenges in supporting these programmes: (i) budget, (ii) human resource development, and (iii) inter-agency coordination and planning for MHW and MHV programmes.

### Budget

In the past, according to the Alien Work Act 2008, migrants were not allowed to work in Thai health facilities. Accordingly, MHWs were employed as labourers or domestic workers to conform with the terms of their work permits. This situation changed in November 2016 with an announcement from the Prime Minister’s Office allowing migrant workers to be legally hired as migrant language coordinators (LCs) under the revised Alien Work Act [20]. Being a LC required only good communication skills in Cambodian, Laotian or Myanmar languages. An important requirement was that LCs had to participate in training programmes approved by the Department of Employment in the Ministry of Labour. In hiring LCs, priority was given to Thai citizens; hiring migrants was only possible if no Thai citizens were available [20]. Thus, after 2016, MHWs were formally recognised as LCs, though the tasks they performed remained unchanged.*“[In the past] jobs allowed for migrant workers were domestic workers and labourers, so in the work permit of MHWs, they were hired only in those two jobs. Nowadays, [we are] allowed to hire [them] as language coordinators.” [HP-9].*

MHWs, especially in public health facilities, complained about low salaries and lack of employment benefits, including sick leave. Although MHWs were permitted to work as LCs there was no regulation of basic salary, salary increments and benefits, because LCs were hired as temporary or project-based contract employees. Discrepancies in remuneration and benefits between public and private sectors influenced competitive recruitment and led to high turnover of MHWs in public health facilities.

*“There is a deduction of salary for sick leave or personal leave. They [MHWs] do not receive these benefits because [the hospital] hires with external contracts [temporary contract]” [HP-14].*

*“The [MHW] salary was 6,900 Baht (US$230) then it was deducted for Social Security Scheme, approximately 300 Baht (10 US$). The remaining allowance was around 6,500 Baht which I had to pay for debts and living costs of the whole families, so it was not enough.” [MHW-7].*

*“[When we announced MHW jobs], there were some unfilled posts because industries paid a higher salary than us [NGOs]. We had to compete with industries which offered minimal remuneration at 12,000-13,000 Baht (US$400). If they [MHWs] passed the probation, they would receive 15,000 Baht (US$500) which was sometimes higher than Thais” [NGO-1].*

In terms of source of funding, migrant workers who have passed the nationality verification processes under the OSS are obliged to be insured with the SSS if they work in the formal sector; while those who work in the informal sectors, including their dependants, are eligible for the HICS [6]. SSS is funded by the government, employers and migrant workers, whereas HICS collects premium payments from migrant workers [6]. HICS revenue is allocated to four categories: health service costs, health promotion and disease prevention costs, high-cost care costs, and administrative costs, including costs of MHW and MHV programmes [21]. For the SSS, the revenue collected is allocated to all general health services at health facilities; but it does not fund specific services for migrant workers [22]. However, 12% of the HICS premium was earmarked for health promotion and disease prevention activities, including the training of MHVs and MHWs [21].

Local interviewees considered the financial support for hiring MHWs, as announced by the MOPH, was not adequate, and that the budget allocation had decreased over time due to a decline in the volume of HICS beneficiaries. Diminishing HICS enrolment was partly due to the implementation of the revised Working of Alien Act in 2017 [23], which stated that employers of migrants must enrol their migrant employees in the SSS, with the exception of specific occupations (mostly jobs in the informal sector such as housemaids and labourers) in which migrants continue to be insured with the HICS.

*“Luckily, we have a high number of migrant workers who enrolled in HICS, so we have enough money to hire MHWs and train both MHWs and MHVs. In some provinces, there is a small number of migrant workers, so it [negatively] affects the budget for MHVs and MHWs.” [HP-5].*

Although falling HICS revenues adversely affected budget support for MHW and MHV programmes, some policymakers addressed that the budget for hiring MHWs and training MHWs and MHVs was manageable in local contexts.

*“The local areas could manage themselves because we [MOPH] supported administrative costs [of HICS revenue]. Some provinces already used the earmarked revenue of the HICS to support health promotion and disease prevention activities, and to hire and train MHWs and MHVs. [POL-1].*

### Human resource development

Human resource development for interpretation and cultural mediation services comprised three elements: a) recruitment, b) training, and c) supervision of MHWs and MHVs. We found variations in the recruitment criteria of MHWs and MHVs in each organisation, related to differences in the responsibilities assigned, which in turn depended on the local context. However, there were some common criteria aligned with national guidelines (see Table 3). For example, MHWs and MHVs should be older than 18, be healthy, have no history of serious illness, drug addiction, mental illness or criminal history, be fluent in their first language and have good communication skills in Thai. They could have Thai or non-Thai nationalities. Interestingly, criteria for educational level and duration of stay in Thailand were not mentioned by interviewees. In some areas, selection criteria specified the need for community engagement. In these instances, most recruits were selected by health staff from MHW and MHV networks.

*“For MHWs, we [health professionals] select migrant workers who have the potential [to be MHVs]. Firstly, they should pass the MHV training course. Secondly, they should speak and write both Thai and Burmese languages. Lastly, they are supposed to have a spirit of volunteerism and a service mind.”* [HP-2].

*“For MHVs, firstly, they should have time to join training courses. Secondly, they should live in those areas, and be well-known persons in the communities. Thirdly, they are supposed to have fairly strong communication skills in Thai because the training programme is in the Thai language, and they have to assist health professionals in communicating with migrant workers.”* [HP-16].

**Table 3:** Recruitment criteria of MHWs and MHVs

Criteria	Migrant health workers	Migrant health volunteers
1. Age	≥ 18 years	
2. Nationality	Thai or non-Thai	
3. Documents	Register with Ministry of Interior or have passport and work permit/letter of consent from employers	Register with Ministry of Interior or have passport/border pass
4. Duration of living in areas	Stay in that area ≥ 1 year and be respected and trusted by migrant workers	Stay in migrant community ≥6 months and selected by ≥10 migrant households and by health staff
5. Attitude	Have “spirit of volunteerism and service mind”	
6. Communication	Be fluent in their first language and have good communication skills in Thai	
7. Role model	Be role model in health and community development	Have leadership, confidence and responsibility
8. Health status	Be healthy, have no serious illness, no drug addiction, mental illness or criminal history	
9. Qualifications	Pass the MHV training courses or have experience in public health	Pass the MHV training courses in other areas
10. Others		Have good employment history and be able to coordinate

Source: Training Curriculum for Migrant Health Workers (2016) [15] and Training Curriculum of Migrant Health Volunteers (2016) [16]

The Nursing Division and Department of Health Service Support in the MOPH developed training manuals with contents and methods that could be adapted to the local context [15, 16]. The main contents of the training manual included basic knowledge on Thai culture, migrant-related laws, different health insurance schemes for migrant workers in Thailand, roles and ethics for MHWs and MHVs, health communication skills, first aid practices, and disease surveillance and health promotion for communicable and non-communicable diseases [15, 16]. The MHV manual was based on the Thai village health volunteer course and manuals, which aimed to promote primary health care and volunteerism [16]. The contents related to common diseases among migrant workers. Some local NGOs developed their own manuals and added content addressing health problems specific to the local context, such as health education on tuberculosis (TB) vigilance, or HIV treatment and prevention. The MHWs’ training programme was more intensive in duration and content than the MHV programme (see Table 4) [15].

*“MHV manuals from NGOs depend on the objective of organisations while in public sectors, [the manual] emphasises health promotion and disease prevention, communicable diseases, roles and responsibilities and basic skills. However, this manual [the national guideline from MOPH] is different from the others as it focuses on the concept of primary health care and volunteerism.” [POL-2].*

**Table 4:** MHW and MHV training courses

Training courses	Migrant health workers	Migrant health volunteers
**Duration**		
Lecture (hours)	40	20
Practice (hours)	80	20
**Contents**		
− Thai culture, laws, health insurance	Yes	Yes
− Roles & ethics − Basic hygiene		
− Essential skills e.g. communication, health assistants		
− Surveillance & prevention of communicable and non-communicable diseases		
− Specific issues e.g. TB and HIV		
− Reproductive health	Yes	No
− Mental health		
− Environmental health		
− Home health care & rehabilitation		

Source: Training Curriculum for Migrant Health Workers (2016) [15] and Training Curriculum of Migrant Health Volunteers (2016) [16]

Some health staff considered that apart from assisting health professionals, MHWs (especially those in public health facilities) should only work as interpreters. In contrast, MHWs considered that they needed to expand their role beyond interpretation to offer health knowledge and skills to patients. Thus, there was a mismatch of expectations between MHWs and health workers.

*“The point is that you [MHW] cannot advise [patients] because that is the role of doctors. You should not tell patients that they have hypertension 100% - you [MHW] can only measure blood pressure and do interpretation.” [HP-3].*

*“Sometimes, they [healthcare staff] performed CPR (cardio pulmonary resuscitation), I would like to learn and know about it. In the emergency situation, it was unpredictable what we would confront, so I would like to help others.” [MHW-7].*

Thai-Myanmar training materials and processes were supported by central MOPH and local organisations; however, local staff could adjust training to the local context. MHWs were trained in the Thai language by local staff from public health facilities and NGOs, while MHVs received bilingual training from both local staff and MHWs. However, some of the MHWs and MHVs interviewed said that they did not fully understand the course contents because of language barriers and time constraints.

*“I [MHV] do not have enough time [to join training courses]. For example, they [local organisations] set up a training programme this week, but I cannot join. When I learn intermittently, it influences our understanding of course contents.” [MHV-3].*

*“Some MHWs cannot understand all the contents [in training courses]. MHWs who clearly understand more than others will sit nearby those who do not understand and explain things to them …*” *[MHW-4].*

MHWs and MHVs were supervised through direct observation by health staff at public facilities and NGOs. Some organizations also used exams to evaluate MHWs’ knowledge, but most were evaluated during on-the-job training. Compared with MHVs, MHWs tended to be more closely supervised during training, because they had longer contact time with staff in public facilities and NGOs during patient consultations.

*“For the supervision of MHWs, when they have activities such as training health knowledge, we [NGO staff] will observe their training. For example, we might ask MHVs whether they understand the training courses or not …” [NGO-3].*

### Interagency coordination and planning

Coordination across organisations was another factor affecting the delivery of the MHW and MHV programmes. We identified a myriad of different mechanisms for collaboration between public health facilities and NGOs. For example, MHWs employed by NGOs served as interpreters in TB and HIV clinics in public hospitals; their services were also called on by public health officers in areas where there was no public provision of MHWs or MHVs. Moreover, MHWs and MHVs were able to join any training course provided by the public sector or NGOs. However, in some provinces, there was a lack of MHV registration data in local organisations. For example, some MHVs trained by the Provincial Health Office were neither registered in the public health facilities nor included in the NGO data. This created problems in human resources management as accurate data on the number and profiles of MHVs were not available.

At the national level, MHW and MHV programmes are one of the key activities in the National Action Plan for Public Health in Specific Areas (Migrant population) 2020–2024 [24]. This plan aims to develop service delivery for migrant workers, migrant health insurance, and intersectoral collaboration [24]. MHW and MHV programs are included in this plan, especially for employment and training [24]. The Bureau of Health Administration and the Department of Health Service Support also support MHW and MHV programmes by providing training guidelines for MHWs, MHVs and health staff, bilingual signage, leaflets, posters and books, and liaises with other sectors for the legalized employment of MHWs as LCs.

*“We [Bureau of Health Administration] develop migrant service delivery … we have YouTube channel, posters in Thai, Myanmar and Laos languages and language manual [Manual Using Four Languages Thai-English-Myanmar-Cambodian For Health Personnel] … in the past, we could not hire [migrant workers as] MHWs, so we changed the laws then they [MHWs] could be hired [as LCs in the work permits]” [POL-3].*

## Discussion

Overall, MHW and MHV programmes in migrant-friendly health services have received much attention from the Thai Government. The Thai healthcare system benefits hugely from the work of MHWs and MHVs, either as interpreters or as cultural mediators. Over the last two decades, there have been significant improvements to the MHW and MHV programmes, such as better training programmes and the provision of an appropriate legal framework for MHWs. However, some challenges still exist, such as the lack of standardisation of training curricula for interpretation services and cultural mediation, insufficient budget to hire MHWs, and a lack of inter-agency coordination and legal basis for overall services.

The concept of health equity is vital in the health system [25]. Migrants’ entitlement to health insurance and health services is key to ensure access to care [25]. According to the MIPEX framework, health systems can aim to be ‘migrant friendly’ by responding to migrants’ needs without necessarily expanding entitlements to equal those of citizens [7]. In Thailand, ‘migrant-friendly health services’ aim to respond to unmet needs and improve migrant access to a level comparable to Thai citizens [12]. For example, the benefit packages in the HICS generally cover all key burdens of diseases in the migrant population, such as communicable diseases and maternal and child health services but there are still some services excluded compared with citizen benefits [21]. The current policy goal of migrant health services in Thailand is to achieve equity with citizen services in general. Along the path to reach equity in migrant health services, Thailand has made great efforts to ensure responsive service delivery for migrants, by implementing the MHV and MHWs programmes and attempting to minimize language barriers between health staff and migrant patients. 

We found much diversity in the way interpretation services and cultural mediation were provided. The MIPEX Health Strand classifies interpretation services as a way to improve the responsiveness of services to migrants’ needs, and cultural mediation as a way to make services more accessible [7]. A study by Phelan and Martin (2010) found that the roles of medical interpreters and cultural mediators can be complementary. For example, cultural mediators help migrant workers to access health services and navigate the system, while medical interpreters come into action when communication barriers become evident [14]. Fluent linguistic skills are a precondition of effective mediation between health personnel and migrants [13].

Though MHWs were initially defined as interpreters and MHVs as cultural mediators, in practice, their roles intermingled. Although it is important to distinguish the roles for the purposes of training and supervision, some managerial flexibility should exist because of the limited number of MHWs and MHVs available. According to local need, MHWs may sometimes perform the function of MHVs and vice versa.

Interpreters are usually classified into two types: formal and informal. Formal interpreters are specially trained for the job, particularly on the technicalities of medical interpretation, cultural sensitivities, and ethical considerations. Informal interpreters are usually ‘ad-hoc’ and may be family members, relatives or friends with knowledge of the language, or bilingual health staff who have other main duties [26]. There are differing views on the value of formal versus informal (or ad-hoc) interpreters. Some migrants perceive that when using family members as interpreters, they feel a high level of trust and do not feel dominated in comparison with using formal interpreters [27]. On the other hand, the use of family members can be problematic because patients may not feel comfortable discussing symptoms in front of them [27]. Moreover, using informal interpreters tends to increase communication errors and misdiagnosis as it depends on the interpreters’ language ability and medical literacy. For these reasons, formal interpreters may be preferable in healthcare settings. Formal interpretation services can increase service utilisation, improve clinical outcomes, and lead to higher satisfaction among migrant patients [28]. Besides, some migrants may prefer professional interpretation over the informal kind because professional interpretation is more likely to ensure the confidentiality of the consultation [27].

Evidence suggests that a contextual approach is advisable, paying attention to health providers’ and patients’ specific needs [29]. For example, some patients may prefer informal interpreters whom they trust, while health professionals prefer formal interpreters (especially during emergencies) due to the necessity of accurately diagnosing and treating life-threatening conditions under time constraints. Providing guidelines to justify the use of formal or informal interpreters in specific situations for health professionals is therefore recommended [30].

In Thailand, MHWs have key roles as formal interpreters in health facilities, while MHVs take on this role when MHWs are not available. Accordingly, MHVs sometimes serve as interpreters for migrant patients; health professionals tend to prefer this to using ad-hoc interpreters like family members and friends. However, neither MHW and MHV curricula offer specific detail about developing interpretation skills such as guidelines for professional ethical conduct (confidentiality, and impartiality) or training for medical interpretation. Strictly speaking, MHVs are not supposed to work as formal interpreters, but in practice they do so when no MHW is available. The number of MHWs is limited, and it would be desirable to appoint more of them and develop their interpretation skills in order to increase the effectiveness of health services.

**Fig. 1:**
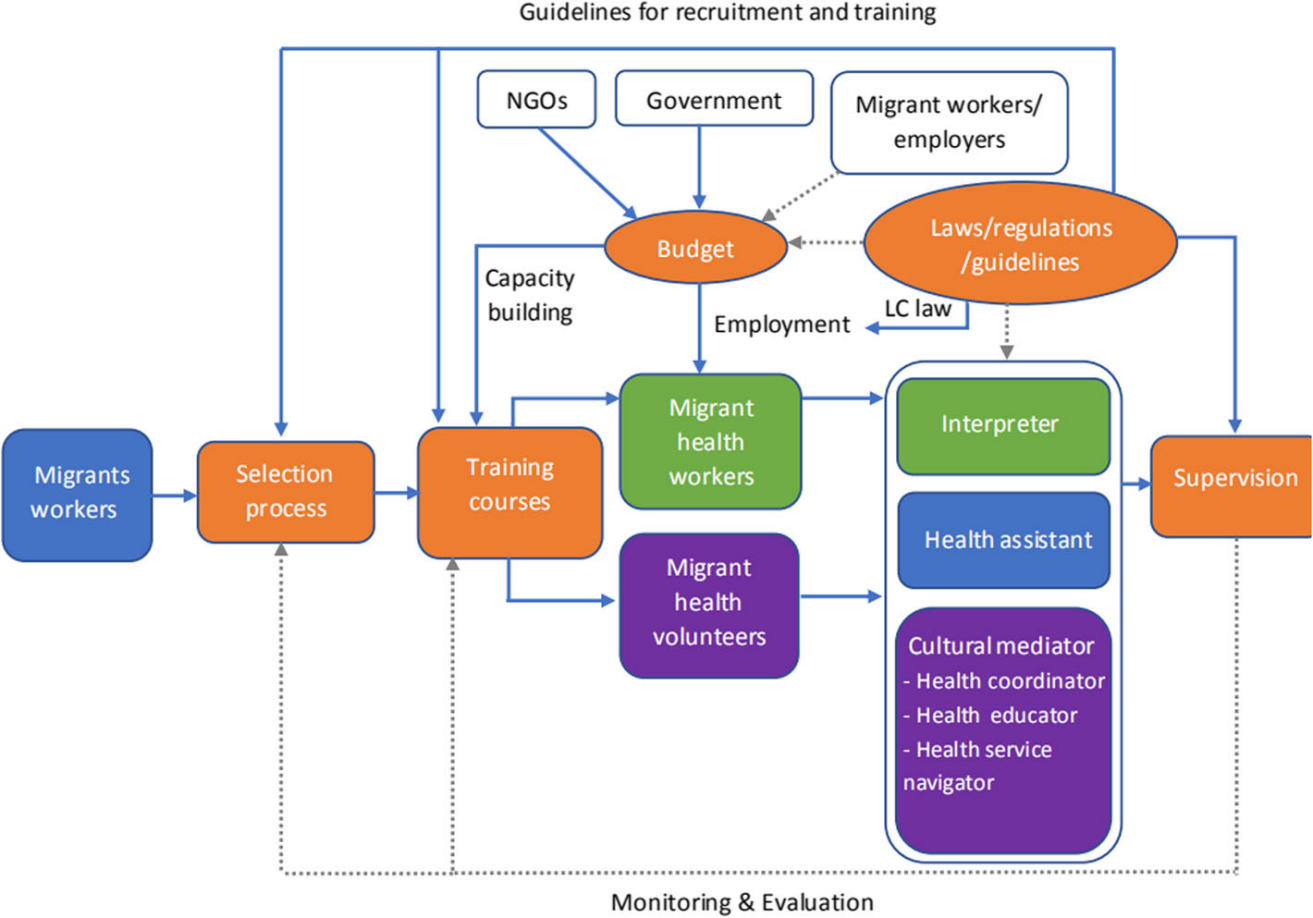
System support for healthcare interpretation services for migrant workers in Thailand. Note: Blue lines refer to flow of work in MHW and MHV programmes; grey line refers to the ideal system supports

Figure 1 provides an overview of the MHW and MHV programmes in Thailand and system support in terms of budget allocation, human resources, and governing mechanisms (orange boxes). Budgetary resources are allocated for the training programmes and MHW employment. Human resources interventions consist of recruitment and selection processes, training courses and supervision. Finally, laws, regulations and guidelines regulate and guide service delivery (examples are the Act permitting MHWs to be hired as LCs, or the use of national guidelines for training and budget support under MOPH regulations).

The roles of MHWs and MHVs combine interpretation and cultural mediation, but there are primary roles defined for each position (green boxes for MHWs and purple boxes for MHVs). However, the system faces many challenges in implementing the MHW and MHV programmes, and ideal resolutions are shown as grey lines in Fig. 1. Ideally, interpretation services and cultural mediation at public facilities and with migrant communities should be supported by laws, regulations and guidelines. Moreover, this governance should ensure that an adequate and sustainable budget is allocated to support these programmes. Migrant workers and employers can also contribute to supporting these programmes. Finally, there should be a feedback loop for MHWs and MHVs from supervision to training and selection process to adjust the work processes into the right direction.

Challenges remain in sustaining these migrant health services; including budget constraints, non-standardized training methods and lack of legal provision to sustain the MHW and MHV programmes. Examples of some challenges follow below.

Firstly, financing is of critical concern as these services can add significant cost to routine services. Each country has different budgetary sources to support interpreter services; for example, most of the intercultural mediators in hospitals in Belgium are funded by the federal government, while in Malaysia, an informal interpreter system is funded and implemented by NGOs [11, 13]. Cultural mediators can be employed by individual migrant workers, NGOs, host institutions like hospitals or municipalities or placement agencies [13]. The conditions of employment vary from being salaried employees, to free-lancers and even volunteers; most contracts are short-term and not considered as professional positions [13].

Even though the cost of using a trained interpreter is higher than using an untrained one, formal interpretation has been claimed to be a more cost-effective and efficient way to improve service utilisation, compliance and health outcomes [31]. More importantly, this service should be free for migrant patients, to reduce the burden of out-of-pocket healthcare expenditure. Some countries have applied technology assisted interpretation, for example, ‘Intercultural mediation through the internet’ and ‘video-remote intercultural mediation’ have been provided in Belgium [13]. In Thailand, interpreter services are provided for free, but the sustainability of this service within the limited budget remains a challenge.

Although the government and NGOs allocate budget to support the employment and training of MHW and MHV programmes, there are still challenges in terms of long-term planning. Owing to the decreasing number of HICS members, the budget available for these programmes has decreased. Moreover, unclear budget allocations and policies affect the remuneration and benefits for MHWs and MHVs, making their employment in the public sector less competitive compared to the private sector, which results in a high turnover of staff. The MOPH should clearly announce the budget allocated for MHW programming in terms of employment and training nationally in addition to the NGO budget. Migrants and employers can contribute to supporting these programmes by allocating funds from SSS to the MHW and MHV programmes.

Secondly, Thailand recruits interpreters through an informal social network of migrants, while interpreters in other countries are recruited from interpretation agencies to ensure quality [32]. Another issue concerning MHWs and MHVs is the engagement of migrant workers as part of the health system. There are some debates on the recruitment of interpreters and cultural mediators with similar backgrounds as migrant workers. However, ethnic matching to ensure the sharing of linguistic and cultural backgrounds does not necessarily guarantee good communication [14]. Concerning cultural mediation, it is necessary to improve health workers’ and migrants’ understanding of each other’s non-verbal communication, meanings and cultures: this requires cultural competence and mediation skills [14], but ethnic matching is neither a necessary nor a sufficient condition for these. Importantly, both Thai and non-Thai people can apply to be MHWs and MHVs, as specified in the guidelines [15, 16].

Guidelines for selecting MHWs and MHVs have not been standardised to ensure the quality of interpreters, and therefore the recruitment process might directly affect the competence of interpreters. For example, there is a difference in quality when using interpreters recruited from the general pool of migrant workers as compared to interpreters who have health professional backgrounds. There is also room for improvement by standardising training programmes. Thailand has attempted to lay down standardised training guidelines for MHWs and MHVs, but these standards do not yet cover training guidelines for interpreters, while quality assurance of the interpretation services is not ensured.

There is no formal state provision for interpreters in healthcare facilities in the other Southeast Asian migrant destination countries, Malaysia and Singapore. In Malaysia, NGOs have implemented informal interpreter systems in their own clinics. For example, refugees are usually hired as interpreters on stipends, as they cannot legally work in the country; the hiring NGO then provides training on professional ethics, patient confidentiality, and medical terminology. Health workers have adopted their own strategies including Google Translate, hand gestures and asking migrant patients to bring a companion as an interpreter. As reported in other settings, medical errors, inaccurate diagnosis and lack of informed consent can occur in the absence of trained interpreters [11, 33].

In terms of training processes, the standard programmes should be clearly defined to ensure that the core capacities of interpreters and cultural mediators continue to improve. For interpreters, three main components exist in the standards of practice (interpretation, cultural interface and ethical behaviour), which are addressed by the International Medical Interpreters Association [34]. On the other hand, for informal interpreters in the UK, Germany and Turkey, training programmes have tackled interpretation skills such as terminology and discourse and promoted a better understanding of ethical and cultural issues among trainees [32]. For cultural mediation, the training programme needs to develop specific knowledge and skills in communication and mediation [14]. Although there are training courses for MHWs and MHVs in Thailand, these courses do not comprehensively cover interpretation skills, especially for translations of medical terminology, and for addressing any ethical and cultural concerns which may arise. Therefore, it is necessary to provide training courses which cover these essential skills in each role.

A study of an MHV programme by Sirilak et al. (2013) revealed that MHV programme management faced challenges including inadequate selection, training and supervision of MHVs, and argued that such programmes needed extra support from the MOPH and international donors. Since 2012, there has been some progress in developing standard guidelines for training MHWs and MHVs. However, problems remain, especially in training and supervision processes. For example, in the above-mentioned study one-third of MHVs did not understand or only partially understood the contents of the training programmes [18]. Similar to the findings of the present study, it showed that MHVs have difficulties understanding course content because of language barriers and inadequate time to attend whole courses.

Moreover, the earlier study found that the supervision system was unclear, resulting in inappropriate monitoring and supervision systems for evaluating the impact of the MHV programme. Although the present study found supervision processes in place in many organisations, they were not systematically conducted and integrated into training plans, representing an example of policy stagnation in MHV programming.

Finally, there should be an element of obligation in the use of interpreters and cultural mediators with migrants [35]. Patients’ rights have become central in many countries, and there can be no informed consent if patient and doctor do not understand each other. Under Italian law, healthcare settings are required to use interpreters when there are language barriers [32]. Although Thailand supports the employment of LCs, there is no regulation or guideline to ensure the use of these services when needed.

Moreover, MHW and MHV programmes are based on MOPH policy pre-announcements, which have no legal authority to compel stakeholders to continue the programmes. Thai labour laws focus mostly on high-skilled foreign professionals. MHWs were not recognised as professional workers and MHVs were, in practice, volunteer-based. The lack of a legal basis to support the work of MHWs and MHVs hence undermines intersectoral coordination and leads to doubts about the sustainability of MHW and MHV programmes.

Using the Hogwood and Gunn (1984) framework of factors affecting policy implementation, there are myriad challenges in MHW and MHV programme implementation, in terms of resources, processes and direction of programmes [36]. There is insufficient budget in some areas and a shortage of workers, depending on the policies of local organisations. MOPH and NGOs now allocate resources for MHW and MHV programmes, which is a good start, but there are no provisions to ensure standardization and the quality of interpreters. For example, training guidelines covering recruitment, training contents and methods, and supervision are all voluntarily implemented. Moreover, the direction of the programme is not based on the needs of stakeholders. This problem aggravates unclear policies in resource management and the sharing of responsibility of each organization (see Table 5).

**Table 5:** Factors affecting policy implementation of MHV and MHW programmes in Thailand

Factors that influence effective implementation of policies	MHW and MHV programmes
No insurmountable external constraints	Both public sector organisations and NGOs follow legislation regulating LC employment; however, the training guidelines are voluntary implemented.
Adequate time and sufficient resources	There is an insufficient budget in some areas and a low number of MHWs and MHVs due to high turnover rate and lack of competitiveness with private sectors.
Requires combinations of resources	The budget allocated to MHW and MHV programmes in public health facilities depends on local agreements and the number of registered migrant workers.
Valid theory	The training courses are not specific to the core competences of interpreters and cultural mediators.
Causal connections are reasonable, clear and direct	It is a good start that MOPH and NGOs allocate resources for MHW and MHV programmes, but it is not well-developed in terms of resource sharing because the programmes started to provide services before resources were properly organised.
Dependency relationships are minimal	It is still unclear which agency has the authority to manage MHW and MHV national programmes.
Understood and agreed objectives	All organisations recognise the importance of MHWs and MHVs, but there are different perspectives on the tasks involved, e.g. some expect MHWs to have only interpretation tasks while some expect them to expand their role beyond this.
Correct sequence of tasks	There is a lack of working processes in the overall system.
Communication and coordination	There is unclear communication about the purposes of the allocated budget, e.g. no specific budget for MHW employment.
Compliance	There is no resistance from health sectors, but some resistance from citizenship privileges and other ministries e.g. employing MHWs in similar fashion as Thai employees and allowing MHWs to receive same benefits as Thai employees are still matters of debate.

Our recommendations for improving the implementation of MHW and MHV programmes are as follows: 1) The MOPH should be a key actor, building partnerships in close cooperation with stakeholders both inside and outside the health sector; 2) All relevant stakeholders should set the objectives and goals of the MHW and MHV programmes, differentiating the roles of MHWs and MHVs while aligning training programmes; 3) All relevant key actors should take part in the monitoring and evaluation of MHWs and MHVs and participate in the tracking of the impact of the programme, and; 4) All policies should be clearly communicated to stakeholders at all levels.

This study provides a system analysis for MHW and MHV programmes, describing how these services are currently provided and resourced, with recommendations to support improvements in the implementation of these programmes. However, the study has some limitations. Firstly, a limited number of study sites means that findings showed only few aspects of service implementation in Thailand. Secondly, health professionals interviewed may be susceptible to social desirability bias in providing the answers that they thought the research team, comprised of health professionals, would like to hear. Nonetheless, these findings provide a necessary update to the 2013 evaluation of the MHV programme at a crucial time where Thailand’s migrant health policies are evolving [18].

## Conclusion

MHW and MHV initiatives are two of the most prominent applications of the concept of migrant-friendly health service policies, implemented as one of the measures to reduce language and cultural barriers between health personnel and migrant workers. MHWs mainly work as interpreters in public facilities and migrant communities, while MHVs work voluntarily as cultural mediators, bridging the gap between health services and their migrant users. Despite the huge merits of the programme in mitigating barriers to healthcare access among migrants, challenges remain in programme implementation. These include insufficient budget support, diverse and unstandardised training courses, and the lack of a legal basis to sustain these initiatives. To ensure the sustainability of MHW and MHV services, the following should be put in place: clear policy regulations on the different roles of MHW and MHV relative to health professionals, standardised training courses including recruitment, training and supervision processes, and efficient financial support from the central government. Moreover, it is important to institute regular monitoring and evaluation of the quality of interpretation services, as well as the entire MHW and MHV programmes. Finally, there should be a lead agency which has the authority to coordinate stakeholders and plan for the overall structure and resource allocation of the MHW and MHV programmes.

## Data Availability

Not applicable.
